# Improving Hemophilia Care in Low- and Middle-Income Countries: Addressing Challenges and Enhancing Quality of Life

**DOI:** 10.7759/cureus.62817

**Published:** 2024-06-21

**Authors:** Adela Perolla, Bledi Kalaja

**Affiliations:** 1 Internal Medicine/Hematology, Mother Teresa Hospital, Tirana, ALB; 2 Hematology, Mother Teresa Hospital, Tirana, ALB

**Keywords:** hemophilia, personalized treatment, education and awareness, caregiver support, mental health support, pain management, prophylactic treatment, comprehensive care, low- and middle-income countries, quality of life

## Abstract

Hemophilia, a genetic bleeding disorder caused by a deficiency in clotting factors, impacts millions of people worldwide. The quality of life (QoL) for those affected remains particularly suboptimal in low- and middle-income countries (LMICs). This article delves into the unmet needs in hemophilia care and management in LMICs, spotlighting various challenges and potential strategies for improvement. One of the primary challenges in LMICs is the limited access to comprehensive care which includes a multidisciplinary approach involving hematologists, physiotherapists, psychologists, and social workers. In many LMICs, the healthcare infrastructure is insufficient to provide such integrated services, leading to fragmented care and poorer health outcomes for individuals with hemophilia. Another significant issue is the challenge of prophylactic treatment. Prophylaxis, which involves regular infusions of clotting factor concentrates to prevent bleeding episodes, is the standard of care in high-income countries. However, in LMICs, prophylactic treatment is often not feasible due to the high cost and limited availability of clotting factor concentrates. This results in a reliance on on-demand treatment, which only addresses bleeding episodes as they occur and does not prevent the long-term complications associated with frequent bleeds. Pain management is another critical area with significant gaps. Chronic pain is a common issue for individuals with hemophilia due to repeated joint bleeds leading to joint damage. In many LMICs, access to effective pain management strategies, including both pharmacological and non-pharmacological treatments, is limited. Mental health support is also a crucial yet often overlooked aspect of hemophilia care. The chronic nature of the condition, combined with frequent hospital visits and the physical limitations imposed by the disease, can lead to mental health issues such as anxiety and depression. However, mental health services are frequently under-resourced in LMICs, and there is a lack of awareness about the mental health needs of individuals with hemophilia. Caregiver support playing a crucial role in managing the day-to-day needs of individuals with hemophilia, is another vital component of hemophilia care that is often insufficient in LMICs. Education and awareness about hemophilia are also lacking in many LMICs. There is often a limited understanding of the condition among the general public and even within the medical community, leading to misdiagnoses and delayed treatment. Employment and financial support are critical issues as well. The physical limitations and frequent medical needs associated with hemophilia can make it difficult for individuals to maintain stable employment, leading to financial strain. In many LMICs, social support systems are inadequate to address these challenges. Lastly, the integration of telehealth and digital health technologies presents a promising strategy to overcome some of these challenges providing remote access to specialist care, education, and support, which is particularly valuable in regions where healthcare resources are scarce. By adopting a multifaceted approach that involves collaboration between governments, healthcare systems, international organizations, and patient advocacy groups, it is possible to address these challenges and significantly improve the QoL for individuals with hemophilia in LMICs.

## Introduction and background

Hemophilia is a genetic disorder that impairs the body's ability to make blood clots, which are necessary to stop bleeding. This condition is typically inherited and is caused by mutations in the genes responsible for producing certain clotting factors, proteins that work together to form a blood clot [1]. It is usually inherited in an X-linked recessive pattern, meaning the faulty gene is located on the X chromosome. Males, having one X and one Y chromosome, are more likely to be affected because they lack a second X chromosome that could potentially carry a normal version of the gene [2,3].

Hemophilia A and Hemophilia B are the two main types, caused by deficiencies in clotting factors VIII and IX, respectively. While advancements in treatment modalities, such as prophylactic clotting factor infusions and gene therapy, have markedly improved outcomes in high-income countries (HICs), the situation remains dire in low- and middle-income countries (LMICs). In these regions, inadequate healthcare infrastructure, high costs of treatment, and limited access to comprehensive care contribute to significant disparities in health outcomes for individuals with hemophilia [1,4].

The World Federation of Hemophilia (WFH) has provided detailed insights through its Annual Global Survey about the current state of hemophilia care globally. The latest data from the 2022 survey highlight contributions from 125 national member organizations, showing the identification and treatment of over 454,690 people with bleeding disorders globally. This survey offers valuable information for understanding and addressing the challenges of hemophilia care worldwide [1].

Hemophilia is a chronic condition that requires lifelong management. In HICs, comprehensive care centers provide multidisciplinary support, including regular prophylactic treatment, physiotherapy, psychological support, and education. This integrated approach significantly enhances the quality of life (QoL) for patients by preventing spontaneous bleeding, maintaining mobility, reducing pain, and empowering patients with knowledge and psychological resilience. QoL refers to the general well-being of individuals, encompassing physical, mental, and social health, including freedom from pain, ability to perform daily activities, emotional stability, and social interaction [5]. However, the scenario is vastly different in LMICs, where healthcare systems often lack the necessary resources and infrastructure to provide similar levels of care [6].

In LMICs, several key challenges hinder the effective management of hemophilia. One of the primary issues is the limited access to clotting factor concentrates, which are essential for both prophylactic and on-demand treatment. The high cost of these products makes them unaffordable for many healthcare systems and patients in these regions [7,8]. Consequently, patients often rely on on-demand treatment, which is less effective in preventing the long-term complications associated with frequent bleeding episodes [9].

Another significant barrier is the lack of awareness and education about hemophilia, both among healthcare providers and the general population. This leads to delayed diagnoses, inappropriate management, and stigmatization of patients. Additionally, the absence of comprehensive care centers means that patients do not receive the multidisciplinary support necessary to manage the various aspects of the condition effectively [10-12].

Pain management and mental health support are also critical areas where LMICs fall short. Chronic pain due to joint damage and the psychological burden of living with a chronic condition are often inadequately addressed [13]. Caregivers, who play a crucial role in the daily management of hemophilia, also lack the necessary support and education to perform their duties effectively.

Despite these challenges, there are promising strategies that can be employed to improve hemophilia care in LMICs. These include increasing the availability of affordable clotting factor concentrates, enhancing education and awareness, integrating Telehealth and digital health technologies, and fostering collaborations between governments, healthcare systems, international organizations, and patient advocacy groups [14].

This review will provide a comprehensive analysis of the current state of hemophilia care in LMICs, identify the unmet needs and challenges, and propose evidence-based strategies for improving the QoL for individuals with hemophilia in these regions.

## Review

The search strategies used for this review involved comprehensive database searches to identify relevant studies on hemophilia in LMICs. The databases searched included PubMed, Scopus, Web of Science, and the Consensus app. The search terms and keywords used were a combination of the following "Hemophilia” and “low- and middle-income countries,” “hemophilia care,” “LMICs,” “Employment support,” “Financial support,” “Mental health,” “Pain management,” and “Education and awareness.” No date or language restrictions were applied during the initial search to ensure comprehensive coverage of the available literature. The searches were conducted up until December 2023. The inclusion criteria for selecting studies or literature sources were studies involving individuals with hemophilia in LMICs, studies examining interventions related to employment support, financial assistance, mental health, pain management, and education/awareness programs, studies reporting on QoL, employment outcomes, financial stability, psychological well-being, pain reduction, and educational outcomes and all types of study designs, including randomized controlled trials (RCTs), cohort studies, case-control studies, cross-sectional studies, and qualitative studies. Our study excluded all the studies involving hemophilia patients in high-income countries only, studies that do not address the specified interventions related to hemophilia care, studies that do not report relevant outcomes, and also studies with insufficient data or unclear methodologies.

Individual studies included in the review were critically appraised for risk of bias using standardized tools appropriate for their design. For RCTs, the Cochrane Risk of Bias tool was employed. For observational studies, the Newcastle-Ottawa Scale (NOS) was used. Qualitative studies were assessed using the Critical Appraisal Skills Program (CASP) checklist.

The findings from the included literature were summarized and synthesized using a narrative synthesis approach. This involved grouping findings into themes such as employment support, financial assistance, mental health, pain management, and education/awareness programs, comparing results within each theme to identify common patterns, discrepancies, and the strength of evidence, and also integrating quantitative and qualitative findings to provide a comprehensive overview of the interventions' impacts on individuals with hemophilia in LMICs. Where applicable, descriptive statistics were used to summarize quantitative data from the studies.

Limited access to comprehensive care

In numerous LMICs, access to comprehensive hemophilia care - encompassing diagnosis, treatment, and rehabilitation - is significantly constrained. This issue arises from insufficient healthcare infrastructure, a shortage of adequately trained healthcare professionals, and inadequate financial resources allocated to hemophilia management [4].

First, strengthening healthcare systems through policy reforms and capacity building is crucial. This includes enhancing the infrastructure of healthcare facilities, expanding the workforce with specialized training programs, and securing sustainable funding for hemophilia care services. Increasing investment in hemophilia care is essential, potentially involving both national funding and international aid. Fostering international partnerships can play a significant role in improving access to essential resources. Collaborative efforts with global health organizations, NGOs, and donor countries can facilitate the provision of necessary medical supplies, support the establishment of treatment centers, and promote knowledge exchange [15].

Furthermore, community-based initiatives can be instrumental in reaching underserved populations [1,10,16]. These initiatives might involve training community health workers to provide basic hemophilia care and education, extending the reach of healthcare services to remote areas [1]. Telemedicine approaches also offer a viable solution to bridge the gap in access to care, particularly for rural populations. Telehealth services can provide remote consultations, follow-up care, and patient education, thereby reducing the need for travel and making specialized care more accessible [14].

Community health worker training programs and telemedicine initiatives can extend the reach of specialized care, while international partnerships can facilitate the provision of resources and knowledge exchange [6]. These strategies collectively enhance the availability and quality of hemophilia care in LMICs, ultimately improving patient outcomes and QoL. Implementing these solutions requires a collaborative effort from governments, healthcare systems, international organizations, and patient advocacy groups to ensure that individuals with hemophilia in LMICs receive the care they need [7,8].

Prophylactic treatment challenges

Prophylactic treatment with clotting factor concentrates is widely regarded as the gold standard for managing severe hemophilia. However, in LMICs, the availability of these treatments is often severely limited, leading patients to rely on on-demand treatments or blood transfusions. Expanding access to affordable prophylactic treatments is vital for enhancing the QoL in these regions [7-9].

One key initiative addressing this issue is the WFH's Humanitarian Aid Program [10], which has made significant strides in providing support. However, ensuring sustainable access to these essential treatments remains a significant challenge. To achieve long-term improvements, a multifaceted approach is necessary.

Local manufacturing of clotting factor concentrates can significantly increase their availability and affordability. By developing production capabilities within LMICs, dependency on international suppliers can be reduced, and costs can be lowered. For example, implementing low-dose prophylaxis in regions like South India has shown marked clinical benefits, with reduced bleeding rates, hospitalization, and improved joint health among children with severe hemophilia [7,17-19].

Fostering public-private partnerships can play a critical role in this endeavor. Collaborations between governments, private sector entities, and NGOs can mobilize resources, share expertise, and create sustainable supply chains [15]. For instance, public-private partnerships in Iran have successfully implemented low-dose escalating prophylaxis regimens, significantly reducing the annual bleeding rate and hospitalization days for children with severe hemophilia [18].

Moreover, policy interventions and financial investments are crucial to support the development and distribution of prophylactic treatments. Governments and international bodies need to prioritize hemophilia care in their health agendas, allocating funds and creating supportive regulatory environments for local production and distribution [9]. In India, studies have demonstrated that low-dose prophylaxis is cost-effective and improves clinical outcomes compared to on-demand therapy. One practical example is the use of low-dose prophylaxis, which is feasible and effective in resource-limited settings [19]. In Kerala, India, children with severe hemophilia who were previously treated with on-demand therapy transitioned to low-dose prophylaxis. This change significantly reduced bleeding rates, hospitalization days, and school absenteeism, thereby improving overall QoL [5,7].

Additionally, efforts to integrate immune tolerance induction (ITI) regimens in LMICs have shown promise. In Tunisia, a low-dose ITI regimen proved effective in managing hemophilia with inhibitors, indicating that even resource-limited settings can adopt advanced treatment protocols successfully [8].

Community-based initiatives and telemedicine can also bridge the gap in access to care. Training community health workers to provide basic hemophilia care and education can extend the reach of healthcare services to remote areas [20]. In China, low-dose secondary prophylaxis has significantly reduced joint bleeding and improved joint function and QoL among children with hemophilia [19].

Personalized treatment barriers

Personalized treatment techniques for hemophilia can greatly improve patient outcomes. However, in LMICs, access to genetic testing and individualized care remains limited [15]. Overcoming these barriers requires a concerted effort from both governments and international organizations.

Investments in diagnostic capabilities are essential to improve access to genetic testing [21,22]. Governments should prioritize the development of genetic testing infrastructure, ensuring that these services are affordable and widely available. Increasing genetic testing capacity will enable more accurate diagnosis and tailored treatment plans, essential for optimizing patient care [22].

In addition to improving diagnostic capabilities, there is a need to implement advanced therapies, such as gene therapy and novel non-factor replacement therapies. Gene therapy offers the potential for a long-term solution by addressing the underlying genetic cause of hemophilia. Meanwhile, novel non-factor replacement therapies provide alternative treatment options that can be tailored to individual patient needs, offering improved efficacy and reduced treatment burdens [21]. 

International organizations can play a pivotal role by providing funding, expertise, and support for the implementation of these advanced therapies in LMICs. Public-private partnerships can also facilitate the introduction of innovative treatments by combining resources and knowledge from various stakeholders [1,2].

Solutions for Personalized Treatment Barriers

Investment in diagnostic capabilities: Governments should invest in genetic testing infrastructure to make these services affordable and accessible. This will enable precise diagnosis and the development of tailored treatment plans [2].

Implementation of advanced therapies: Introducing gene therapy and novel non-factor replacement therapies can provide long-term solutions and personalized care. These therapies address the root cause of hemophilia and offer more effective treatment options [2,21].

International collaboration: International organizations can support LMICs by providing funding, expertise, and logistical support for the implementation of advanced therapies. Public-private partnerships can enhance resource mobilization and knowledge sharing [1,2].

Policy and financial support: Governments and international bodies should prioritize hemophilia care by allocating funds and creating supportive regulatory environments for the local production and distribution of treatment products [1].

Pain management gaps

Chronic pain, predominantly resulting from joint damage, is a prevalent issue among individuals with hemophilia. In LMICs, the options for managing this pain are often limited, significantly affecting daily activities and overall QoL [5,23]. Addressing this challenge requires the development of effective and affordable pain management strategies, incorporating both pharmacological and non-pharmacological interventions.

Pharmacological interventions should include access to appropriate pain relief medications tailored to the needs of hemophilia patients. This involves ensuring the availability of analgesics and anti-inflammatory drugs that are both effective and safe for long-term use. Studies indicate that access to oral morphine in LMICs can be significantly improved through structured programs like the American Cancer Society's Treat the Pain program, which has successfully expanded the availability of affordable oral morphine in several African countries [23].

Non-pharmacological approaches are equally important and should encompass a range of multidisciplinary strategies. Physical therapy can help maintain joint function and reduce pain through tailored exercise programs. Occupational therapy can assist individuals in adapting their daily activities to manage pain better and maintain independence. Psychological interventions, such as cognitive-behavioral therapy (CBT) and hypnosis [24], have been shown to significantly improve pain management and QoL in hemophilia patients. For instance, an RCT demonstrated the effectiveness of hypnosis in reducing pain interference and promoting health-related QoL among people with hemophilia [5,23].

Furthermore, integrating these multidisciplinary approaches into a cohesive pain management plan can significantly enhance the overall care for hemophilia patients in LMICs. This integration requires a concerted effort to improve healthcare infrastructure and training for healthcare providers. Effective pain management in hemophilia should also include patient education on self-management techniques. A qualitative study in Iran highlighted the importance of cognitive and spiritual strategies for pain relief and self-management in hemophilia patients [25].

Mental health support

The psychological burden of living with hemophilia, including anxiety, depression, and social isolation, is often overlooked in LMICs. Addressing these mental health challenges is crucial for improving the overall QoL for individuals affected by hemophilia [5,25].

Providing access to mental health services is a fundamental step. This includes ensuring the availability of professional psychological support, such as counseling and therapy, tailored to the needs of hemophilia patients. Establishing support groups can also offer significant benefits by providing a platform for individuals to share experiences, gain emotional support, and reduce feelings of isolation [26].

Educational resources are essential for both patients and their families, helping them understand the psychological aspects of hemophilia and equipping them with strategies to manage mental health issues effectively. These resources should be accessible and culturally appropriate to ensure they meet the diverse needs of the population [27].

Cultural and community-based approaches to mental health support can address the unique challenges faced by people with hemophilia in LMICs. Integrating mental health services within the existing community health frameworks can enhance their acceptability and effectiveness. For example, training community health workers to provide basic mental health support and refer patients to specialized services can bridge the gap in mental health care [28].

Additionally, leveraging community resources and traditions can enhance mental health interventions. Engaging local leaders and using culturally relevant practices can facilitate greater acceptance and utilization of mental health services [29].

Solutions for Mental Health Support

Access to professional psychological support: Governments and health organizations should ensure the availability of counseling and therapy tailored to the needs of hemophilia patients. Establishing support groups can also help reduce social isolation [26].

Educational resources: Providing culturally appropriate educational resources for patients and families to understand and manage mental health issues effectively [27].

Community-based approaches: Integrating mental health services within community health frameworks and training community health workers to provide basic support and referrals to specialized services [28].

Leveraging cultural resources: Engaging local leaders and using culturally relevant practices to enhance the acceptance and utilization of mental health services [28].

Implementing these strategies will improve the emotional well-being and QoL of individuals with hemophilia in LMICs.

Support for caregivers

Caregivers are indispensable in the lives of people with hemophilia, yet they frequently endure substantial emotional, financial, and physical burdens. Supporting caregivers is crucial for enhancing the QoL for both individuals with hemophilia and their caregivers. Implementing comprehensive support strategies in LMICs can significantly alleviate these burdens [30].

Educational Programs

One key strategy is to provide educational programs tailored to caregivers. These programs can equip caregivers with essential knowledge about hemophilia management, treatment options, and emergency care procedures. By enhancing their understanding and skills, caregivers can provide better care and feel more confident in their roles [1].

Respite Care Services

Respite care services are another vital component of caregiver support. These services offer temporary relief to caregivers, allowing them to rest and recharge. Respite care can be provided through community health services, NGOs, or government programs, ensuring caregivers have regular opportunities to take breaks without compromising the care of their loved ones [31].

Financial Assistance

Financial assistance is also crucial, given the economic strain that caregiving can impose. Providing financial support, such as subsidies or grants, can help alleviate the financial burdens associated with medical expenses and loss of income due to caregiving responsibilities [30].

Emotional Support and Counseling

Access to support groups and counseling services can significantly improve the emotional well-being of caregivers. Support groups offer a platform for caregivers to share their experiences, receive emotional support, and learn from others in similar situations. Counseling services can provide professional psychological support to address stress, anxiety, and depression commonly experienced by caregivers [32].

Integrated Caregiver Support Strategies

By implementing these strategies-educational programs, respite care, financial assistance, and emotional support-LMICs can substantially improve the QoL for caregivers of individuals with hemophilia, thereby positively impacting the overall care and well-being of the patients [1,5,30].

Educational programs: Tailoring educational programs to caregivers can enhance their knowledge and confidence in managing hemophilia [1].

Respite care services: Offering respite care can provide necessary breaks for caregivers, improving their ability to provide long-term care [30].

Financial assistance: Financial support can alleviate economic burdens, making it easier for caregivers to fulfill their roles without undue stress [33].

Emotional support and counseling: Support groups and counseling services can address the psychological burdens faced by caregivers, improving their overall well-being [33].

Education and awareness

Enhancing public understanding of hemophilia is essential to reduce stigma, improve access to care, and facilitate better social integration for individuals with hemophilia in LMICs. Educational campaigns targeting healthcare providers, schools, and the general public are critical for raising awareness and promoting an accurate understanding of the disorder.

Healthcare providers are often the first point of contact for individuals with hemophilia. Therefore, targeted educational programs for medical professionals can improve diagnosis, treatment, and ongoing care. These programs should include up-to-date information on hemophilia management and the importance of comprehensive care approaches [1].

Schools play a significant role in the lives of children with hemophilia. Educating school staff and students about hemophilia can help create a supportive and inclusive environment. Training sessions for teachers and school nurses can provide practical information on managing hemophilia, recognizing symptoms, and responding to emergencies [33].

Public awareness campaigns are also vital. These campaigns can use various media platforms, including television, radio, social media, and community events, to disseminate information about hemophilia. The goal is to foster a more informed and compassionate society that understands the challenges faced by people with hemophilia and supports their needs [34]. 

Collaboration with local patient organizations and community leaders is crucial for ensuring that educational efforts are culturally relevant and effective. These collaborations can help tailor messages to specific cultural contexts, making them more relatable and impactful. Community leaders can also serve as influential advocates, helping to spread awareness and reduce stigma within their communities [1].

Solutions for Education and Awareness

Targeted educational programs for healthcare providers: These programs should focus on up-to-date information on hemophilia management and comprehensive care approaches to improve diagnosis and treatment [1].

Training sessions in schools: Educating school staff and students can create a supportive environment for children with hemophilia. Training for teachers and school nurses can provide practical information on managing the condition [19].

Public awareness campaigns: Utilizing media platforms to disseminate information about hemophilia can foster a more informed and compassionate society [30].

Collaboration with local organizations and community leaders: Ensuring that educational efforts are culturally relevant and effective by working with patient organizations and community leaders [30].

Employment and financial support

Living with hemophilia often incurs significant costs, and many individuals struggle to maintain employment due to their health challenges. Ensuring access to appropriate employment opportunities and financial support is crucial for improving the QoL for individuals with hemophilia in LMICs [5,33].

Employment Opportunities

Developing programs that help individuals with hemophilia find suitable employment is essential. This may involve job placement services, vocational training, and career counseling tailored to the needs and capabilities of individuals with hemophilia. These initiatives can empower individuals to secure stable employment and achieve financial independence. For instance, comprehensive care models have demonstrated significant improvements in employment rates among individuals with hemophilia [1].

Workplace Accommodations

Ensuring that workplaces are accommodating to the needs of employees with hemophilia is critical. This includes creating flexible work schedules, providing access to necessary medical facilities or breaks for treatment, and educating employers about hemophilia to foster an inclusive work environment. Legal protections and incentives for employers who accommodate employees with hemophilia can also be effective. Such accommodations can help reduce absenteeism and enhance productivity [31].

Financial Assistance

Medical expenses associated with hemophilia can be substantial. Providing financial assistance programs, such as subsidies for clotting factor concentrates, coverage for medical treatments, and disability benefits, can alleviate the economic burden on individuals and their families. These programs can be funded through government budgets, international aid, and partnerships with NGOs. For example, the National Rural Health Mission in India successfully reduced out-of-pocket expenditures for hemophilia patients through government-funded free treatment services [1,20,30].

Policy Development

Governments should develop and implement policies that protect the rights and interests of individuals with hemophilia. This includes anti-discrimination laws, healthcare policies that ensure access to necessary treatments, and social welfare programs that provide economic support. Policies that mandate health insurance coverage for hemophilia treatment can significantly reduce financial strain on patients and their families [1,10].

Job placement and vocational training programs: Developing tailored employment services and vocational training for individuals with hemophilia to secure stable jobs and achieve financial independence [4,6].

Workplace accommodations: Creating flexible work environments and educating employers about hemophilia to foster inclusion and reduce absenteeism [6].

Financial assistance programs: Providing subsidies, medical coverage, and disability benefits to alleviate the economic burden on individuals and their families [1,10].

Policy development: Implementing laws and policies that protect the rights of hemophilia patients and ensure access to necessary treatments [17].

Integration of telehealth and digital health technologies

Integrating telehealth and digital health technologies offers significant potential to improve access to care, patient monitoring, and self-management for individuals with hemophilia in LMICs. Implementing telemedicine services, mobile health applications, and electronic health record (EHR) systems can streamline care delivery and empower patients to manage their conditions more effectively [32,34].

Telemedicine Services

Telemedicine enables remote consultations, allowing patients to receive expert medical advice without the need to travel long distances [34]. This is particularly beneficial for those living in rural or underserved areas, where access to specialized hemophilia care is limited. Telemedicine can facilitate regular follow-ups, emergency consultations, and specialist referrals, ensuring continuous and comprehensive care [34].

Mobile Health Applications

Mobile health applications can support self-management by providing tools for tracking symptoms, medication schedules, and treatment progress. These apps can offer reminders for medication and appointments, educational resources about hemophilia, and direct communication channels with healthcare providers. By using mobile health apps, patients can take a more active role in managing their health, leading to better adherence to treatment plans and improved outcomes [32].

EHR Systems

Implementing EHR systems can enhance the efficiency and coordination of care. EHRs allow healthcare providers to access and update patient information in real-time, ensuring that all relevant medical data is available during consultations. This can improve the accuracy of diagnoses, the effectiveness of treatment plans, and the continuity of care [34].

Ongoing Education and Communication

Digital health technologies can also support ongoing education and communication between patients, caregivers, and healthcare providers. Online platforms and virtual support groups can provide valuable information and emotional support, helping patients and caregivers stay informed and connected. Educational webinars and tele-education programs can keep healthcare providers updated on the latest advancements in hemophilia care, ensuring that patients receive the best possible treatment [32,34].

Telehealth and digital health technologies in LMICs can overcome many barriers to hemophilia care, enhancing access, improving patient outcomes, and empowering individuals to manage their conditions more effectively.

## Conclusions

This review underscores the critical unmet needs and challenges faced by individuals with hemophilia in LMICs. Addressing these issues requires a comprehensive approach, including enhancing healthcare infrastructure, expanding the healthcare workforce, and securing sustainable funding. Increasing the availability of affordable prophylactic treatments through local manufacturing and public-private partnerships is essential. Investing in genetic testing and advanced therapies is vital for personalized treatment. At the same time, effective pain management strategies, combining pharmacological and multidisciplinary non-pharmacological interventions, are necessary to improve QoL. Providing mental health services, support groups, and culturally relevant educational resources is crucial for addressing psychological burdens.

Supporting caregivers with educational programs, respite care, financial assistance, and counseling can alleviate their burdens. Raising public awareness through targeted educational campaigns can reduce stigma and facilitate social integration. Integrating telehealth and digital health technologies can improve access to care, patient monitoring, and self-management. By implementing these strategies, LMICs can significantly enhance the QoL for individuals with hemophilia, fostering a more inclusive and supportive healthcare environment.
